# Corticotropin-releasing hormone receptor-1 antagonist attenuates visceral hypersensitivity induced by trinitrobenzene sulfonic acid colitis and maternal separation in rats

**DOI:** 10.1186/s13030-025-00324-0

**Published:** 2025-03-28

**Authors:** Ryoko Hasegawa, Kumi Nakaya, Motoyori Kanazawa, Shin Fukudo

**Affiliations:** 1 Department of Behavioral Medicine, Tohoku University Graduate School of Medicine, 2-1 Seiryo, Aoba, Sendai, 980-8575 Japan; 2 Division of Epidemiology, School of Public Health, Tohoku University Graduate School of Medicine, Sendai, Japan; 3 Division of Personalized Prevention and Epidemiology Department of Preventive Medicine and Epidemiology Tohoku Medical Megabank Organization, Sendai, Japan; 4 Department of Psychosomatic Medicine, Tohoku University Hospital, Sendai, Japan; 5 Research Center for Accelerator and Radioisotope Science, Tohoku University, Sendai, Japan; 6 Department of Psychosomatic Medicine, Japanese Red Cross Ishinomaki Hospital, Ishinomaki, Japan

**Keywords:** Irritable bowel syndrome, Early - life stress, Maternal separation, Corticotropin-releasing hormone, Visceral hypersensitivity

## Abstract

**Background:**

The prevailing paradigm for the etiology of irritable bowel syndrome is that transient noxious events lead to long-lasting sensitization of the neural pain circuit, despite complete resolution of the initiating event. In this study, we tested the hypotheses that (1) the combination of maternal separation (MS) and previous colorectal inflammation induces extensive visceral hypersensitivity in rats and (2) visceral hypersensitivity induced by maternal separation and previous colorectal inflammation in rats is mediated via the corticotropin-releasing hormone receptor-1 (CRH-R1) pathway.

**Methods:**

Male rat pups were separated from their dams from postnatal day 2 to postnatal day 21. Acute colitis was induced by colorectal administration of trinitrobenzene sulfonic acid (TNBS) or vehicle on postnatal day 8. On postnatal day 50, the visceromotor response was evaluated by electromyography of the abdominal muscle in response to graded (10–80 mmHg) and phasic colorectal distention (CRD) one time. The same experiments were repeated after administration of the selective CRH-R1 antagonist CP-154,526 (20 mg/kg) or vehicle at 45 min before CRD.

**Results:**

Compared with control rats, visceral perception was increased in MS + TNBS rats. MS + TNBS rats showed a significantly larger visceromotor response to phasic CRD with 40 mmHg, 60 mmHg, and 80 mmHg. Compared with vehicle administration in MS + TNBS rats, administration of CP-154,526 significantly attenuated this visceromotor response to CRD with 40 mmHg, 60 mmHg, and 80 mmHg.

**Conclusions:**

These findings suggest that the combination of previous colitis and early life stress induce visceral hypersensitivity, and that the CRH-R1 pathway may play a role in this sensitization.

**Supplementary Information:**

The online version contains supplementary material available at 10.1186/s13030-025-00324-0.

## Background

Developmental plasticity in physiological systems is an important mechanism by which organisms can adapt their physiological responses to environmental demands [1, 2]. Although such adaptations can be beneficial for immediate survival [3], they may also result in permanent alterations in physiological responses to environmental challenges and predispositions to pathological conditions in later life [3–5]. The hypothalamic-pituitary-adrenal (HPA) axis is a neuroendocrine system that is subject to programming by early life events [6, 7], as evidenced by a large number of studies in both humans and animal models [3, 8, 9]. For instance, newborn rats subjected to maternal separation demonstrate increased release of corticotropin-releasing hormone (CRH) [6], decreased expression of hippocampal glucocorticoid receptors [8] due to DNA methylation [9, 10], and changes in the noradrenaline [9] and γ-amino butyric acid systems [11]. Therefore, individual differences in the stress response are at least in part due to early life events.

Irritable bowel syndrome (IBS) is a common disorder characterized by chronic abdominal pain and discomfort that is associated with alterations in bowel habits in the absence of macroscopic pathology [12]. The pathogenesis of IBS is probably multifactorial [12], with the major causes reported as gastrointestinal dysmotility [13], visceral hypersensitivity [14], and psychological disorder [15]. The original view of the disease as a primary disturbance of the gut is being conceptually refined to include a complex and disordered interaction between the brain and gut [16, 17], where the physiological contractions of the gut are perceived as painful by IBS patients [14]. Different types of stressors appear to play key roles in the development of IBS as well as in the modulation and maintenance of the disease throughout life [18–20]. Early life stressors associated with childhood neglect, abuse, loss of a parent, and life-threatening situations during childhood have been shown to increase the risk of developing IBS [18–20]. Coutinho et al. [21] reported that rats exposed to maternal separation showed visceral hyperalgesia and increased colonic motility in response to stress after the development. Al-Chaer et al. [22] demonstrated that colonic irritation in neonatal rats induces chronic visceral hypersensitivity to painful stimuli. Moreover, a prospective study proved that individuals with a high perception of stress are likely to develop IBS after recovering from acute bacterial enterocolitis [23]. Therefore, the prevailing paradigm of IBS etiology is that transient noxious events lead to long-lasting sensitization of the neural pain circuit, despite complete resolution of the initiating event.

CRH is a major mediator of the stress response [24]. It was the first peptide isolated from a family of mammalian corticotrophin-releasing hormone (CRH)-related peptides that now includes urocortin 1, urocortin 2 (also known as stresscopin-related peptide), and urocortin 3 (also known as stresscopin) [25, 26]. CRH plays a major role in controlling the stress response in the colon [27]. As in the case of a number of neuropeptides that act in the brain to influence gut motility, the CRH ligands and receptors that were initially characterized in the brain (where they function to influence gut motor function) have since been shown to be widely expressed in peripheral tissues, including the gastrointestinal tract of experimental animals [28] and humans [29]. In a previous study, our group clearly demonstrated that IBS patients show an exaggerated response to CRH administration [29]. Sensory and motor dysfunctions of the colon in IBS patients are improved by the administration of a CRH antagonist [30]. An earlier study indicated that neonatal trauma induced phenotypic changes in adulthood, including enhanced vulnerability of the gut mucosa to stress via mechanisms involving peripherally located CRH receptors [31]. Another study demonstrated that CRH acts peripherally to stimulate colonic motility and that CRH receptor-1 (CRH-R1) is primarily involved in mediating the colonic motor response induced by CRH and the water avoidance stress test [27]. Given this background, we tested the following hypotheses in the present study that (1) the combination of maternal separation and previous colorectal inflammation induces extensive visceral hypersensitivity in rats; and that (2) visceral hypersensitivity induced by maternal separation and previous colorectal inflammation in rats is mediated via the CRH-R1 pathway.

## Materials and methods

### Animals

Male Wister rat pups at postnatal day 1 (PND 1) and their dams were obtained from Charles River Breeding Laboratories Japan, Inc. (Yokohama, Japan). Ten pups were housed in a standard cage with 1 dam until the weaning period. The dam was given free access to food and water under a 12:12-h light-dark cycle (lights on at 08:00) in an animal room set at a temperature of 23 ± 1 °C. The irritation procedure and experimental testing were conducted during the light component of the cycle.

This study was conducted in accordance with the Guide for the Care and Use of Laboratory Animals and approved by the Tohoku University Environmental & Safety Committee.

### Protocol

**Fig. 1:**
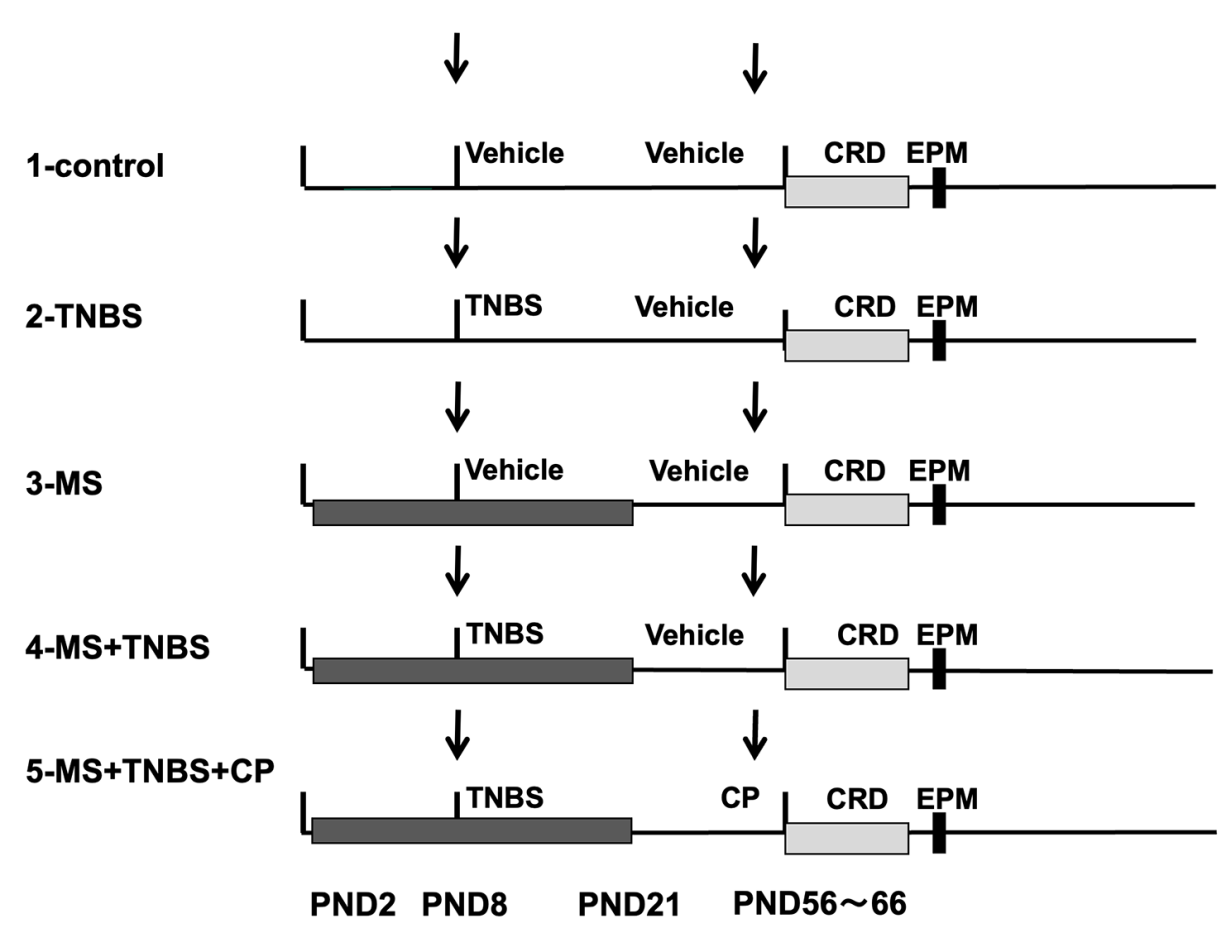
Study protocol. Grouping of rats on the postnatal day (PND); (1) No maternal separation/no inflammation (control); (2) No maternal separation/inflammation (trinitrobenzene sulfonic acid: TNBS); (3) Maternal separation/no inflammation (MS); (4) Maternal separation/inflammation (MS + TNBS); (5) Maternal separation/inflammation/CRH antagonist CP-154,526 (MS + TNBS + CP). Dark gray rectangle: MS at PND2-21, left downward arrow: intracolonic administration of vehicle or TNBS, right downward arrow: subcutaneous injection of vehicle or CP, light gray rectangle: colorectal distention (CRD), black vertical bar: elevated plus maze (EPM)

The animals were assigned randomly to 5 groups **(**Fig. 1**)**. In each of the groups, at least 7 animals were used in each of the following conditions: (1) no maternal separation/no inflammation (control); (2) no maternal separation/inflammation (TNBS); (3) maternal separation/no inflammation (MS); (4) maternal separation/inflammation (MS + TNBS); and (5) maternal separation/inflammation/CRF antagonist CP-154,526 (MS + TNBS + CP). The pups with maternal separation were separated from their dams from PND 2 to PND 21, while the pups without maternal separation remained with their dams. For previous inflammation, the rats were treated with trinitrobenzene sulfonic acid (TNBS; WAKO Pure Chemicals Ltd., Tokyo, Japan) on PND 8. For no inflammation, the rats were treated with vehicle (5% dimethyl sulfoxide [DMSO], 5% Cremophor EL, 90% saline).

Rat abdominal myograms were recorded on PND 47. Colorectal distension (CRD) was performed to assess visceral sensitivity on PND 50. At 45 min before CRD, the rats were treated subcutaneously with the CRH-R1 antagonist CP-154,526 or vehicle. At 30 min after CRD, the rats were exposed to an elevated plus maze (EPM) and their behavior was monitored and quantified. The rats were killed by decapitation at the end of the experiment. At 25 min after the EPM, blood was collected in a tube and plasma was separated by centrifugation at 3000 rpm and stored at -30 °C.

### Maternal separation

Litters of either 8 or 10 pups were assembled randomly for each fostering. The maternal separation protocol was essentially the same as that described in earlier reports [8, 9, 21, 31, 32]. Briefly, the rats were exposed to a 180-min period of daily separation from their dams (MS180) from PND 2 to PND 21. Separation was started at 09:00 ± 30 min each day. The dams were removed from maternity cages and placed into separate identical cages until the end of the manipulation. On removal of the dams, MS180 litters were removed as a group from the nest, weighed, and placed as a group in an isolation cage in an adjacent room. The isolation cage was lined with chip bedding and placed in a neonatal cage kept at 37 ± 0.5 °C using a heating mat (KN–474; Natume, Tokyo, Japan) placed underneath the cage. At the end of the daily separation, the pups were returned to their maternity cages and rolled in the soiled home cage bedding material before being reunited with the dam. On PND 22, all rats were weaned and the litters were housed in pairs (same treatment) in individual cages.

### Previous inflammation

The previous inflammation model of experimental colitis is well documented elsewhere [32, 33]. In brief, TNBS was dissolved in 50% ethanol to a concentration of 120 mg/mL. At PND8 during maternal separation rats were transferred in the paper container for treatment. A polyethylene-60 catheter was inserted at 3 mm past the anus to lie approximately at the level of the splenic flexure. The rats were infused with 25 µL of the TNBS/ethanol solution (0.75 mg TNBS). The control rats were similarly intubated but infused with 25 µL of 50% ethanol only (vehicle). This procedure needed a recovery period of 6 weeks [33].

### Colorectal distention and assessment of visceral sensitivity

The experimental procedure for CRD and assessment of visceral sensitivity were conducted as described previously [34]. Electrodes with plastic-coated stainless steel wire (Star Medical, Tokyo, Japan) were stitched into the external oblique muscle just superior to the inguinal ligament for electromyography (EMG). The electrode leads were then tunneled subcutaneously and externalized at the nape of the neck for future access. Wounds were closed in layers with 4 − 0 silk stitching material. Following surgery, the rats were housed separately and allowed to recuperate for 2 or 3 days. Wounds were checked for absence of tenderness to ensure complete recovery from surgery before testing.

For CRD, the rat was lightly restrained in a plastic tube. A polyethylene balloon of 2.5 cm diameter was inserted into the colorectum via the anus. The distal end of the balloon was positioned at 1 cm proximal to the anus and secured in place by taping the balloon catheter to the base of the tail. Balloon pressure, which represents intracolonic pressure, was continuously monitored online with the aid of a computed barostat system (G and J Electronics, Inc., Toronto, Canada). The colorectum was distended at a pressure of 10, 20, 40, 60, or 80 mmHg for 20 s with an interval of 3 min. The order of stimulus intensity was selected at random.

CRD in rat results in an easily monitored pseudo-affective response that is characterized by contraction of the abdominal musculature (i.e., a visceromotor reflex) [34, 35]. This visceromotor response to CRD was quantified by measuring EMG activity in the external oblique muscle [34]. EMG activity was amplified, filtered (G and J Electronics, Inc., Toronto, Canada), and displayed on a computer screen. A voltage threshold was arbitrarily set so that no potential exceeded it under the basal condition. There was an increase in EMG activity in the external oblique muscle during distention, and the number of spikes crossing the pre-set voltage threshold was counted using a software program [34]. Counts of spikes per second were recorded for 20 s before (baseline), during, and after CRD. An increase in the total number of counts during distention over baseline was taken as a response. The distending pressure and EMG activity were digitized and processed using a software program. During CRD, there was an increase in EMG activity that was time-locked to the stimulus compared with baseline. Baseline responses to graded intensities of phasic CRD (10, 20, 40, 60, and 80 mmHg) were obtained in all rats.

### Elevated plus maze

Anxiety-like behavior was evaluated using a Plexiglas EPM [35, 36]. The four arms of the maze (50 × 10 cm) were located 1 m above the floor with a 10-cm center, and the closed arms had 40-cm high walls. The rats were placed in the center of the maze and allowed to explore the maze freely for 5 min. The behavior of each rat was recorded by video camera and Ethovision software (TARGET system, MS-DOS). The EPM test belongs to a group of unconditioned anxiety models used to evaluate the effects of putative anxiolytic compounds. The paradigm is based on rats’ innate aversion to high open spaces. In this study, the EPM was used at 30 min after the end of CRD in all rats.

### Role of CRH in visceral sensitivity

In the CRD protocol, MS + TNBS + CP rats were injected subcutaneously with the CRH-R1 antagonist CP-154,526 (Pfizer, Inc., Groton, CT) before CRD [27]. The dose of CP-154,526 was set at 20 mg/kg according to the results of an earlier report on the effect of CP-154,526 on stress-induced colonic motility [27]. CP-154,526 was kept at room temperature and dissolved in a mixture of 5% DMSO (Sigma Chemical Co., Tokyo, Japan), 5% Cremophor EL (Sigma), and 90% saline before administration. The other groups were injected subcutaneously with vehicle only at 45 min before CRD.

### Neuroendocrine function

Immediately after the rats were decapitated, blood was collected in chilled polyethylene tubes containing 200 µL (74.5 mg) EDTA and separated. The plasma was stored at -30 °C until assay. Plasma adrenocorticotropic hormone (ACTH) and serum corticosterone were measured by radioimmunoassay.

### Statistical analysis

All values are expressed as mean ± standard error of the mean. A two-tailed Student’s t-test was used to analyze differences between two groups. When more than two groups were compared, the significance among groups was evaluated by one-way analysis of variance (ANOVA), and further statistical post hoc comparisons were performed using a post hoc Tukey’s test. A probability level of < 0.05 was considered to be statistically significant. All statistical calculations were performed using SPSS for Windows (ver. 12.0 J).

## Results

### Influence of maternal separation and TNBS on the body weight

**Fig. 2:**
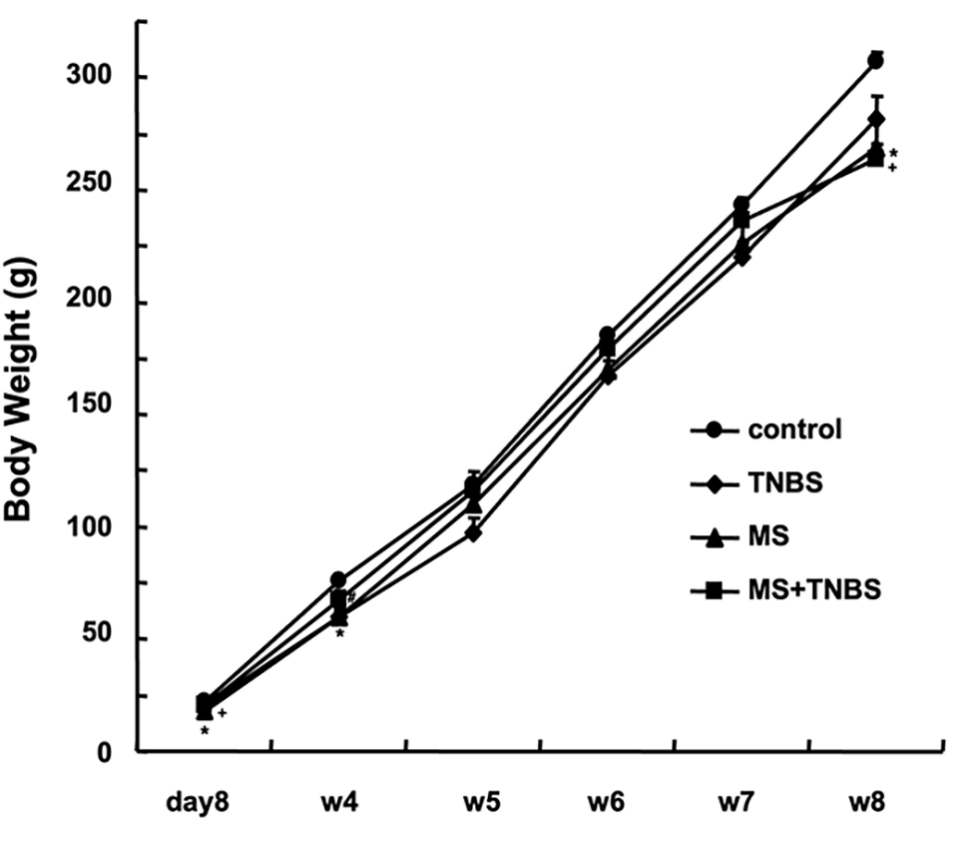
Changes in body weight. Mean body weight (g) with error bar of SD of controls (circle), TNBS (diamond), MS (triangle), and MS + TNBS (square) in the postnatal period. *P < 0.05 MS vs. control, ^+^*P* < 0.05 MS + TNBS vs. control, ^#^*P* < 0.05 TNBS vs. control

Changes in body weight were significantly different among the control, TNBS, MS, and MS + TNBS rats (ANOVA, F_15_ = 1.828, *P* < 0.035). On PND 8, both MS rats (*P* < 0.01) and MS + TNBS rats (*P* < 0.05) weighed significantly less than control rats. At postnatal week 4, MS rats (*P* < 0.01) and TNBS rats (*P* < 0.01) weighed significantly less than control rats. At postnatal week 8, MS rats (*P* < 0.05) and MS + TNBS rats (*P* < 0.05) had gained less weight than control rats **(**Fig. 2**)**.

### Visceral sensitivity

**Fig. 3:**
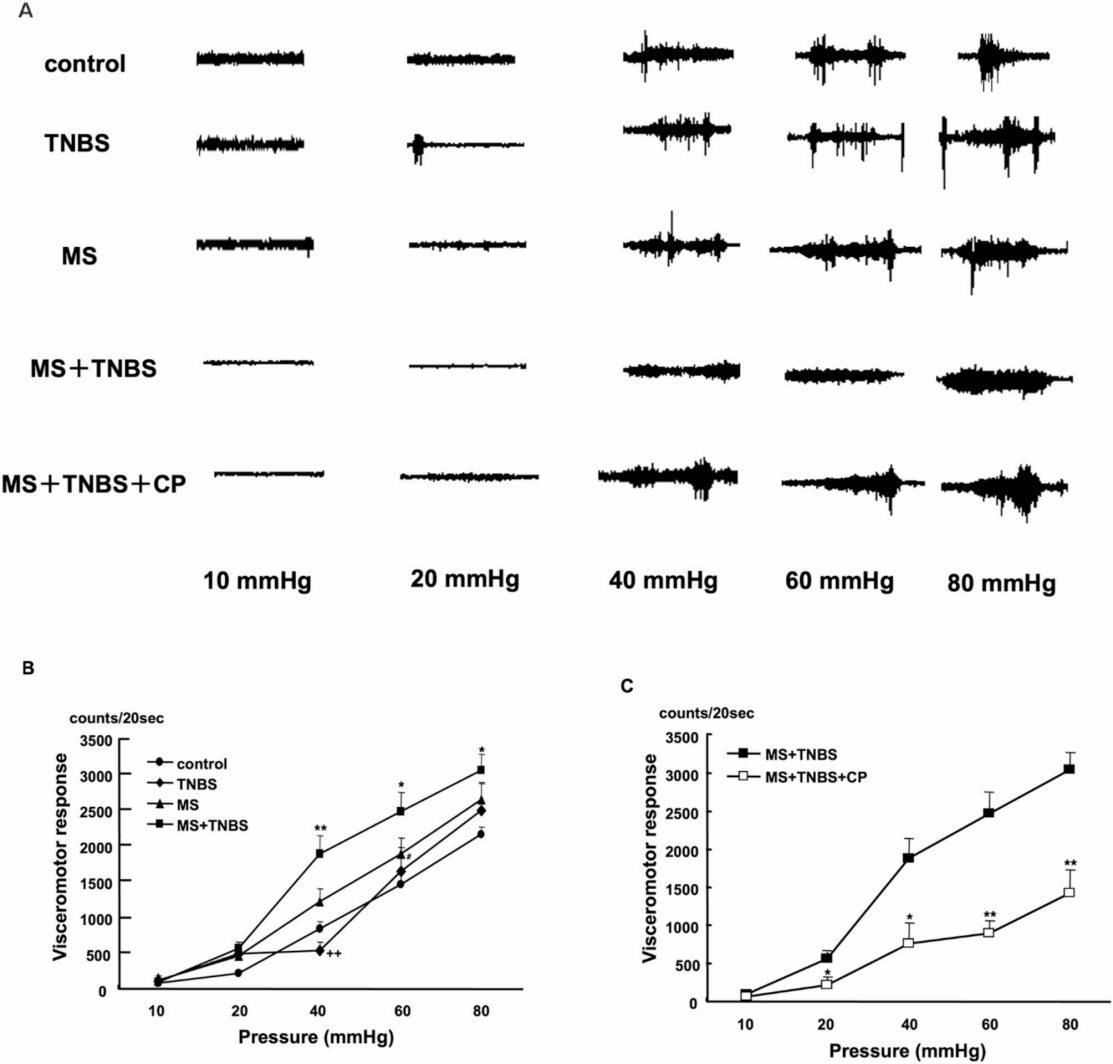
Visceral pain evaluated with visceromotor response. **(A)** Visceromotor response represented as electromyographic activity under graded pressure with 10, 20, 40, 60, and 80mmHg of CRD. **(B)** Difference in visceral sensitivity (counts/20 s) to colorectal distention with 10, 20, 40, 60, and 80 mmHg among all groups. *P < 0.05, **P < 0.01 MS + TNBS vs. controls, ^++^*P* < 0.01 MS + TNBS vs. TNBS, ^#^*P* < 0.05 MS vs. control. **(C)** Difference in visceral sensitivity (counts/20 s) to colorectal distention with 10, 20, 40, 60, and 80 mmHg between MS + TNBS rats and MS + TNBS + CP rats. *P < 0.05, **P < 0.01 vs. MS + TNBS

Responses to graded intensities of phasic CRD were obtained in the control (*n* = 9), TNBS (*n* = 7), MS (*n* = 8), and MS + TNBS (*n* = 9) rats (Fig. 3A). Significantly more spikes per 20 s were observed in a CRD pressure-dependent manner in all rats (ANOVA, F_12_ = 3.139, *P* < 0.001). Compared with the control rats, stimulus-response functions were increased in all other rats; however, this increase was especially remarkable in the MS + TNBS rats. MS + TNBS rats produced a more significant visceromotor response to phasic CRD with 40 mmHg (*P* < 0.01), 60 mmHg (*P* < 0.05), and 80 mmHg (*P* < 0.05) than the control rats **(**Fig. 3B**)**. They were also significantly more sensitive to CRD at 40 mmHg than TNBS rats (*P* < 0.01). There was no difference in visceromotor response between MS + TNBS and MS. MS rats showed a significantly larger visceromotor response at 60 mmHg than the control rats (*P* < 0.05).

### Effect of the CRH-R1 antagonist on visceral hypersensitivity

The administration of 20 mg/kg CP-154,526 before CRD (MS + TNBS + CP) significantly shifted stimulus-response functions to the left compared with the procedure with MS + TNBS (ANOVA, F4 = 8.940, *P* < 0.001). MS + TNBS + CP rats showed a significantly smaller visceromotor response to CRD with 20 mmHg (*P* < 0.05), 40 mmHg (*P* < 0.05), 60 mmHg (*P* < 0.01), and 80 mmHg (*P* < 0.01) than MS + TNBS rats **(**Fig. 3C**)**.

### Maternal separation, previous inflammation, and anxiety-related behavior

There was no significant difference in anxiety-related behavior evoked by the EPM among all rats (Table 1). However, MS + TNBS + CP rats tended to show a greater percentage of time spent in the open arms and more entries into the open arms than MS + TNBS rats.

**Table 1:** Anxiety-related behavior of experimental rats

	%Time Spent in Open Arms	Number of Entries into Open Arms
	Mean ± SE	Mean ± SE
control	14.6 ± 5.4	2.0 ± 0.8
TNBS	15.3 ± 7.4	2.1 ± 0.8
MS	23.6 ± 6.9	2.4 ± 0.6
MS + TNBS	20.7 ± 7.0	3.6 ± 1.1
MS + TNBS + CP	34.6 ± 6.7	4.6 ± 0.8

No significant difference in the percentage of time spent in the open arms or the number of entries into the open arms by the elevated plus maze was found among controls (*n* = 9), TNBS (*n* = 7), MS (*n* = 9), MS + TNBS (*n* = 7), and MS + TNBS + CP (*n* = 7) rats

### Neuroendocrine data

**Fig. 4:**
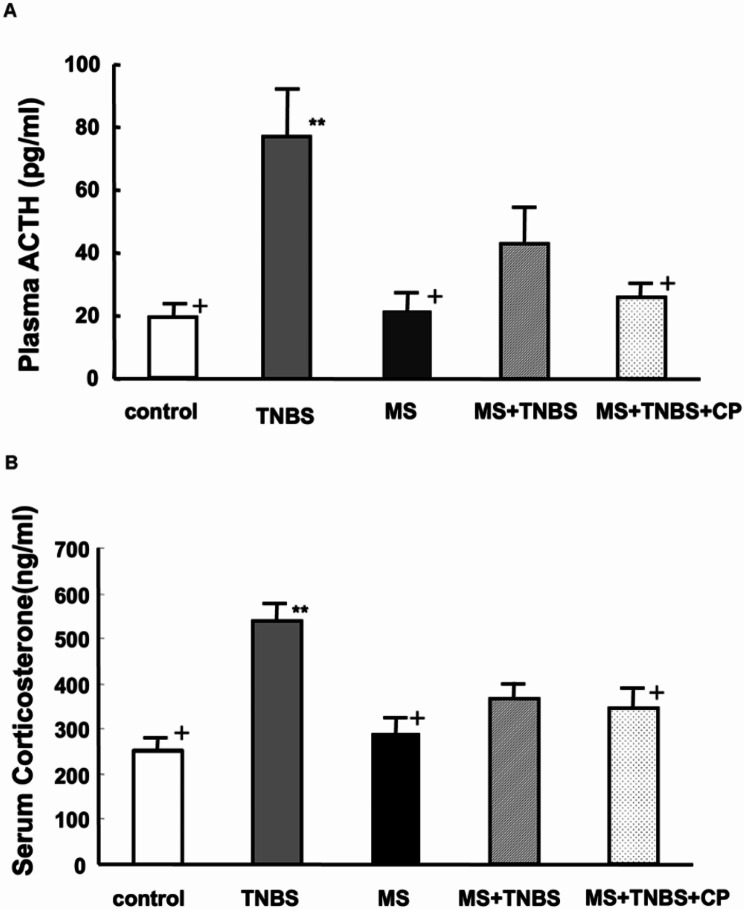
Plasma ACTH and serum corticosterone. **(A)** Difference in plasma ACTH levels among all groups. **P < 0.001 vs. controls, ^+^*P* < 0.05 vs. TNBS. **(B)** Difference in serum corticosterone levels among all groups. **P < 0.001 vs. controls, ^+^*P* < 0.05 vs. TNBS

Plasma ACTH levels were significantly different among the control (*n* = 10), TNBS (*n* = 10), MS (*n* = 8), MS + TNBS (*n* = 8), and MS + TNBS + CP rats (ANOVA, F_4_ = 7.899, *P* < 0.001) **(**Fig. 4A**)**. Plasma ACTH levels were higher in TNBS rats than those in control (*P* < 0.001), MS (*P* < 0.001), and MS + TNBS + CP (*P* < 0.05) rats. There was no significant difference in plasma ACTH among the control, MS, MS + TNBS, and MS + TNBS + CP rats.

Serum corticosterone levels were significantly different among the controls, TNBS, MS, MS + TNBS, and MS + TNBS + CP rats (ANOVA, F_4_ = 9.120, *P* < 0.001) **(**Fig. 4B**)**. TNBS rats showed significantly higher corticosterone levels than controls (*P* < 0.001). There was no significant difference in serum corticosterone levels among the control, MS, MS + TNBS, and MS + TNBS + CP rats (data not shown).

## Discussion

In this study, we found that newborn rats exposed to a combination of neonatal maternal separation-induced stress and previous colorectal inflammation (colitis) developed visceral hypersensitivity in later life. Moreover, our results show that the administration of a CRH-R1 antagonist (CP-154,526) attenuates this hypersensitivity, suggesting that CRH-R1 plays a crucial role in visceral sensitization. Coutinho et al. [21] reported that rats exposed to single neonatal trauma in the form of maternal separation show visceral hyperalgesia and increased colonic motility in response to stress at 2 months of age [21]. In contrast, our data suggest that single maternal separation induces visceral hypersensitivity in a limited way, that is, only at colorectal distention with 60 mmHg. Our data also showed no visceral hypersensitivity after the recovery of TNBS alone with attenuated visceral sensitivity compared with MS + TNBS at 40 mmHg CRD. The difference in the development of visceral hypersensitivity after maternal separation or TNBS between our study and those of Coutinho et al. [21] and Al-Chaer et al. [22] is unclear. However, one explanation could be differences in minor inflammatory states of the colon. Because our study is based on our previous study of recovered inflammation of the colon by the administration of TNBS [34], our method of combined previous inflammation and MS might have some advantages over the earlier studies.

Our results clearly indicate that a combination of early life trauma and previous colorectal inflammation, rather than only a single insult of neonatal trauma, may be a key factor in gut sensitization through CRH-R1. Previous studies have suggested a possible mechanism for visceral hypersensitivity induced by a combination of early life stress and peripheral inflammation. For instance, basic alterations in central neural pathways have been reported after an intense stressful experience in rats [37]. Stress-induced visceral hyperalgesia has been shown to be caused by alterations in dorsal column-mediated pain modulation [37]. Moreover, visceral afferent nerve terminals have been shown to be sensitized by autonomically mediated changes in target cells in the colon following acute stress and mast cell degranulation [38]. The activation of enterochromaffin cells has also been suggested to play a role in this sensitization [12]. Shanks et al. [39] reported that exposure of neonatal rats to a low dose of endotoxin results in long-term changes in HPA axis activity with elevated mean plasma corticosterone concentration due to increased corticosterone pulse frequency and pulse amplitude. In addition to these effects on the HPA axis, exposure of neonatal rats to endotoxin had long-lasting effects on immune system regulation, including increased sensitivity of lymphocytes to stress-induced suppression of proliferation and remarkable protection against adjuvant-induced arthritis. Collins et al. [33] reported that at 6 weeks after the administration of TNBS to rats, stress caused a significant increase in myeloperoxidase activity as compared to stressed controls, even though plasma corticosterone levels were similar between the two groups. Previous colitis has been shown to render the colon more susceptible to the effects of stress on enteric nerve function and to increase inflammation parameters in response to stress [33]. These findings together with our present results support the notion of a potent and long-term effect of neonatal exposure that can induce major changes in the development of both neuroendocrine and immunological regulatory mechanisms.

The immune environment during development not only alters inflammatory and neuroendocrine responses throughout life, but also modifies predisposition to stress-related pathological conditions via HPA activation [39]. These alterations have also been reported in IBS patients, namely previous bacterial enterocolitis [23], minor inflammation [40], traumatic stress in early life, and altered adrenocorticotropic hormone responses to CRH [29]. In the present study, the administration of the CRH-R1 antagonist CP-154,526 attenuated visceral hypersensitivity in rats with maternal separation and previous colitis. Therefore, CRH and the CRH-R1 pathway may play a crucial role in visceral hypersensitivity induced by a combination of early life stress and previous colorectal inflammation. The precise sites of action of the CRH-R1 antagonist in this study are unknown; however, CRH immunoreactivity and CRH-R1 are reported to be abundant in the cerebral cortices and myenteric plexus [41, 42]. Both sites are crucial for the processing of visceral perception. We found in a previous rat study that administration of CRH-R1 antagonist into the central nucleus of the amygdala attenuated visceral nociception and noradrenaline release from the amygdala [43]. It is therefore plausible that CRH and the CRH-R1 pathway may, at least in part, contribute to the maintenance of gut sensitization after colitis and early life stress.

## Strength

The strength of our study is that reduced body weight in the rats with maternal separation after colitis is consistent with previous findings. Maternal separation or previous colorectal inflammation initiates a complex bio-behavioral response, including decreased secretion and suppression of cell responses to trophic hormones, such as growth hormone and insulin, leading to growth retardation [2–5].

## Limitation

The first limitation is that there was no significant difference in anxiety-like behavior and ACTH/corticosterone levels among the controls, maternally separated rats, maternally separated rats after colitis, and CRH-R1 antagonist-treated maternally separated rats after colitis. Because the effects of CRH and the CRH-R1-mediated reaction on gastrointestinal function are not always parallel to anxiety-like behavior and ACTH/corticosterone levels [28], our results are justified. The second limitation is that only rats after the TNBS colitis showed increased ACTH and corticosterone levels. We had, in fact, predicted increased ACTH and corticosterone release in rats with maternal separation based on an earlier study [10]. This finding could be explained by the altered expression of other ACTH secretagogues in rats after colitis. Many studies have demonstrated that the mother-pup interaction, as a key regulator of HPA axis neuroplasticity during the first postnatal weeks, is very important [44, 45]. In this vulnerable period, handling of pups is reported to cause attenuation of HPA axis responsivity due to inhibition of CRH production in the paraventricular nucleus of the hypothalamus [46]. In the present study, the handling time in the pups for maternal separation may have been more than that in TNBS-treated rats, during this vulnerable period of nerve plasticity. Therefore, novel severe inflammation with shorter handling time might sensitize the responsivity of the HPA axis in newborn rats. The third limitation is that only male rats were examined. We examined both male and female rats in our previous study [32]. By contrast, we focused on male rats in this study because accurate female experiments need evaluation of the menstrual cycle. This design is similar to many animal experiments [47], but further examination of female rats is needed. Furthermore, other neural circuits including CRH-R2, serotonin, noradrenaline, dopamine, glutamate [12], and oxytocin [48] may also be involved in visceral hypersensitivity after early life trauma and previous colorectal inflammation.

## Conclusions

In conclusion, our results justify the two hypotheses put forward in this study: (1) the combination of maternal separation and previous colorectal inflammation induces extensive visceral hypersensitivity in rats; and (2) visceral hypersensitivity induced by maternal separation and previous colorectal inflammation in rats is mediated via the CRH-R1 pathway. These findings suggest that CRH-R1 receptors may be useful targets for the treatment for patients with post-infectious IBS with early childhood adversity.

## Electronic supplementary material

Below is the link to the electronic supplementary material.

Supplementary Material 1

Supplementary Material 2

## Abbreviations

CRH: Corticotropin-releasing Hormone
CRD: Colorectal distention
EPM: Elevated plus-maze
IBS: Irritable bowel syndrome
MS: Maternal separation
PND: Post-natal day
TNBS: Trinitrobenzene-sulfonic acid
