# Supplementary material for: Corticotropin-releasing hormone receptor-1 antagonist attenuates visceral hypersensitivity induced by trinitrobenzene sulfonic acid colitis and maternal separation in rats
Source: Biopsychosoc Med. 2025 Mar 28;19:5. doi: 10.1186/s13030-025-00324-0 (PMC11951537; doi:10.1186/s13030-025-00324-0)
Supplement: Supplementary file 1 — Supplementary Material 1 [file 13030_2025_324_MOESM1_ESM.pdf]

Shin Fukudo, MD, PhD
Research Professor
Research Center for Accelerator and Radioisotope Science
Tohoku University
2-1 Seiryo, Aoba, Sendai 980-8575, Japan
Phone & FAX +81-22-717-8214
E-mail address: shin.fukudo.c6@tohoku.ac.jp

January 16, 2025

Dear Editor, Biopsychosocial Medicine

Enclosed please find our secondly revised manuscript **"A Corticotropin-Releasing Hormone Antagonist Attenuates Visceral Hypersensitivity Induced by Trinitrobenzene Sulfonic Acid Colitis and Maternal Separation in Rats" BPSM-D-24-00068.R2** by **Hasegawa R et al.**, for consideration for publication in Biopsychosocial Medicine.

Thank you for your editorial judgement as potentially acceptable for publication in Biopsychosocial Medicine. By way of your e-mail on January 16, 2025, we understood that we should revise our manuscript due to the English edition by the reviewer #4. We performed it and updated the manuscript. We very much appreciate the great effort of editors and reviewers to find scientific significance of our study. We hope that the second revision of the manuscript is now worth for publication in Biopsychosocial Medicine.

Sincerely,

Shin Fukudo, MD, PhD
