# Supplementary material for: Corticotropin-releasing hormone receptor-1 antagonist attenuates visceral hypersensitivity induced by trinitrobenzene sulfonic acid colitis and maternal separation in rats
Source: Biopsychosoc Med. 2025 Mar 28;19:5. doi: 10.1186/s13030-025-00324-0 (PMC11951537; doi:10.1186/s13030-025-00324-0)
Supplement: Supplementary file 2 — Supplementary Material 2 [file 13030_2025_324_MOESM2_ESM.docx]

**Authors’ Response to Reviewers**

**Reviewer #1:** The authors took the reviewers' comments seriously and adequately addressed each reviewer's questions, adding new supportive logical arguments to the manuscript.

**Authors’ Response:** Thank you for your very nice suggestion.

**Reviewer #2:** Authors have appropriately addressed my comments. I have no further comments.

**Authors’ Response:** Our paper has been improved by your scientific insight.

**Reviewer #3:** Excellent revision.

**Authors’ Response:** Thank you for your comments and correction in detail.

**Reviewer #4:** Although your paper was especially well written, I have made a few suggestions for change that I hope you find helpful.

**Editor:** Please check the comments by Reviewer #4 to improve English expressions, the native editor of the journal.

**Authors’ Response:** We very much appreciate to your great effort on English expression for publication of our manuscript. English in our manuscript has been definitely improved and now it is worth for publication.
